# HIV Prevalence and Associated Risk Factors among Individuals Aged 13-34 Years in Rural Western Kenya

**DOI:** 10.1371/journal.pone.0006470

**Published:** 2009-07-31

**Authors:** Pauli N. Amornkul, Hilde Vandenhoudt, Peter Nasokho, Frank Odhiambo, Dufton Mwaengo, Allen Hightower, Anne Buvé, Ambrose Misore, John Vulule, Charles Vitek, Judith Glynn, Alan Greenberg, Laurence Slutsker, Kevin M. De Cock

**Affiliations:** 1 Division of HIV/AIDS Prevention, U.S. Centers for Disease Control and Prevention (CDC), Atlanta, Georgia, United States of America; 2 Kenya Medical Research Institute (KEMRI)/CDC, Kisumu, Kenya; 3 KEMRI/Institute of Tropical Medicine (ITM), Kisumu, Kenya; 4 Institute of Tropical Medicine (ITM), Antwerp, Belgium; 5 Republic of Kenya Ministry of Health, Kisumu, Kenya; 6 Kenya Medical Research Institute, Kisumu, Kenya; 7 London School of Hygiene and Tropical Medicine, London, United Kingdom; 8 Division of Parasitic Diseases, U.S. Centers for Disease Control and Prevention (CDC), Atlanta, Georgia, United States of America; 9 Global AIDS Program, U.S. Centers for Disease Control and Prevention (CDC), Atlanta, Georgia, United States of America; Lerner Research Institute, Cleveland Clinic, United States of America

## Abstract

**Objectives:**

To estimate HIV prevalence and characterize risk factors among young adults in Asembo, rural western Kenya.

**Design:**

Community-based cross-sectional survey.

**Methods:**

From a demographic surveillance system, we selected a random sample of residents aged 13-34 years, who were contacted at home and invited to a nearby mobile study site. Consent procedures for non-emancipated minors required assent and parental consent. From October 2003 - April 2004, consenting participants were interviewed on risk behavior and tested for HIV and HSV-2. HIV voluntary counseling and testing was offered.

**Results:**

Of 2606 eligible residents, 1822 (70%) enrolled. Primary reasons for refusal included not wanting blood taken, not wanting to learn HIV status, and partner/parental objection.

Females comprised 53% of 1762 participants providing blood. Adjusted HIV prevalence was 15.4% overall: 20.5% among females and 10.2% among males. HIV prevalence was highest in women aged 25-29 years (36.5%) and men aged 30-34 years (41.1%). HSV-2 prevalence was 40.0% overall: 53% among females, 25.8% among males. In multivariate models stratified by gender and marital status, HIV infection was strongly associated with age, higher number of sex partners, widowhood, and HSV-2 seropositivity.

**Conclusions:**

Asembo has extremely high HIV and HSV-2 prevalence, and probable high incidence, among young adults. Further research on circumstances around HIV acquisition in young women and novel prevention strategies (vaccines, microbicides, pre-exposure prophylaxis, HSV-2 prevention, etc.) are urgently needed.

## Introduction

African youth are disproportionately affected by the global HIV pandemic. Of 10 million HIV-infected persons aged 15-24 years, 62% live in sub-Saharan Africa; the majority are female. [1] The 2007 Kenya Aids Indicator Survey (KAIS) demonstrated a national HIV prevalence of 7.4% among Kenyans aged 15-49 years; 8.8% in females and 5.5% in males. [2] Among adolescents aged 15-19 years, HIV prevalence was 3.5% in females and 1.0% in males, increasing to 7.4% and 1.9% respectively among those aged 20-24 years. [2] Nyanza Province in western Kenya continues with Kenya's highest HIV prevalence of 15.0%; 17.6% in women and 11.4% in men. [2] The KAIS HIV prevalence data varied slightly, a non-statistically significant difference, from the 2003 Kenya Demographic Health Survey (KDHS). [3]

In Nyanza Province, the Kenya Medical Research Institute (KEMRI) and the U.S. Centers for Disease Control and Prevention (CDC) have conducted infectious disease research since 1988 in Asembo, a rural community. [4] An ongoing demographic surveillance system (DSS) in this area reports a life expectancy at birth of 38 years and high adult mortality rates likely attributable to AIDS. [5] In 2002 the Institute of Tropical Medicine in Antwerp, Belgium (ITM), with KEMRI/CDC, initiated a multi-component adolescent prevention intervention in Asembo. This intervention was in response to a needs assessment, conducted by ITM and KEMRI/CDC among Nyanza youth, and to findings from a 1997-1998 study [6] demonstrating an HIV prevalence of 23% and 3.5% among females and males aged 15-19 years, respectively, in Kisumu, Nyanza's capital. [7] Little was known about HIV epidemiology in rural areas, where the majority of Nyanza's population resides.

In 2003, KEMRI, CDC, ITM, and the London School of Hygiene and Tropical Medicine (LSHTM) initiated the first HIV-specific study in Asembo to measure the prevalence of HIV and sexually transmitted infections (STIs), identify factors associated with HIV infection, provide a baseline to evaluate a new adolescent prevention intervention, and inform sample size calculations for a prospective HIV-incidence cohort. We present HIV and HSV-2 prevalence and associated factors for 13-34 year old Asembo residents.

## Methods

### Ethics Statement

Before study initiation, ethical approval was obtained from the Kenya Medical Research Institute (KEMRI), the U.S. Centers for Disease Control and Prevention (CDC), the Institue of Tropical Medicine (ITM), and the London School of Hygiene and Tropical Medicine (LSHTM) Ethics Review Committees/Institutional Review Boards.

### Sampling

Asembo is a rural, subsistence farming community in Nyanza. The entire population of approximately 65,000, over 95% of whom are Luo, participates in an ongoing DSS maintained by KEMRI/CDC. [5] Asembo is comprised of compounds or clusters of houses occupied by family members. DSS staff visit each compound every four months to collect demographic data and register all births, deaths, and in-, out-, and trans-migrations. [5]

Based on Kisumu data from Buvé *et al*, [7] we chose a study population age range of 13-34 years. Using the DSS as a sampling platform, potential study participants were randomly selected through stratified sampling by sex and age group. First, compounds were randomly selected from a DSS list of compounds housing residents aged 13-34 years. Second, one individual per compound aged 13-34 years was randomly selected for participation, through randomly assigned numbers, to minimize potentially non-independent HIV transmission. For analyses, individual sample weights were calculated to account for differences in sampling fractions by the 8 sex- and age-strata (females: 13-15, 16-19, 20-29, and 30-34 years; males: 13-18, 19-24, 25-29, and 30-34 years) which included adjustments for nonresponse. These weights were used for all analyses labeled as being weighted. For sample size and power calculations, we assumed similar HIV prevalence rates to those in Kisumu. [7] Allowing for acceptable precision estimates in the sex- and age-strata with the highest anticipated HIV prevalence plus a migration/refusal rate of 25%, 3960 individuals were randomly selected and approached for study participation.

### Consent Procedures for Adults and Minors

Written informed consent was obtained from all adults and mature minors before study participation. Minors (<18 years of age) were classified as “mature” or “non-mature” using legal definitions. Mature minors were married, pregnant, or a parent and could consent to study participation, as they can for HIV voluntary counseling and testing in Kenya. [8] Non-mature minors required a two-step written consent-assent process. A parent/guardian was asked for written informed consent for their minor to participate in the study. After parental/guardian consent was given, private discussion and thorough explanation of the study to the minor followed, and the minor was asked to provide written informed assent to study participation.

### Data/Specimen Collection Procedures and Provision of Test Results

Data were collected from October 2003 through April 2004. Trained interviewers visited compounds to obtain informed consent from potential study participants. Participants were invited to a nearby site on a specified day for the interview and specimen collection. Study clinicians conducted physical examinations to diagnose acute illnesses or symptomatic STIs and confirm male circumcision.

All participants underwent pre-test HIV counseling by certified counselors before venipuncture. Participants could obtain their HIV results and post-test counseling confidentially through study counselors at community health facilities. Adult participants were encouraged to share their HIV results with sexual partners and minors with their parent/guardian.

### Provision of Services

Participants received free clinical care for common, acute ailments including STIs and were referred for free tuberculosis diagnosis and treatment. Sexual partners of participants with STIs were offered free STI treatment. HIV care and support services were provided through existing infrastructure developed collaboratively between the Ministry of Health and CDC's Global AIDS Program.

### Laboratory Procedures

Initially, the HIV testing algorithm used Determine (Abbott Laboratories, Tokyo, Japan) and Unigold (Trinity Biotech Plc. Bray, Ireland) rapid tests in parallel. Discordant results were resolved with Capillus (Trinity Biotech Plc. Bray, Ireland). Concordant negatives were tested with the sensitive Murex enzyme immunoassay (Abbot, Dartford, UK). Negative Murex results were considered negative for HIV-infection. All Murex HIV-positive samples were further run on Western blot (Genelab Diagnostics, Belgium) and read according to CDC criteria. [9]

For efficiency and cost, halfway through the study we changed to parallel testing by ELISA using Vironostika HIV Uniform II plus O kit (Organon Teknika, Boxtel, the Netherlands) and Enzygnost (Dade Behring, Marburg, GmbH, Germany). Concordant results were final. Discordant results were run on Western blot (Genelab Diagnostics, Belgium). No statistical adjustments were made with this change in the HIV testing algorithm because a number of systematically selected specimens that had been tested with the original HIV testing algorithm were retested with the second algorithm with consistent results.

HSV-2 was screened using an HSV-2 type-specific IgG ELISA (Kalon Biological, Ltd., Surrey, UK) with a reported sensitivity of 92.3% and specificity of 97.7% in African populations. [10]

Urine pregnancy tests were conducted on-site for all consenting females not visibly pregnant using Randox, Inc. latex monoclonal agglutination test (Antrim, Northern Ireland, UK) and First Sign HCG One Step (UNIMED International, Inc., South San Francisco, CA, USA).

### Data management and Analyses

Data were collected on optical character recognition enabled forms that were completed in the field through face-to-face interviews, transported to the research station, scanned into a database using Teleform version 8 (Verity, Inc., 2003, Sunnyvale, California, USA), and exported into Access 2000. Questionnaires contained embedded internal consistency and validity checks. Data cleaning and statistical analyses using survey procedures were performed in SAS versions 8.2 through 9.13 (2003-2005, SAS Institute, Inc., Cary, North Carolina, USA) and STATA 8.2 (StataCorp LP, 2003, College Station, Texas, USA).

HIV prevalence and univariate aggregate data were weighted to the population from which the study sample was drawn. Standard errors were computed that accounted for the sampling design and the individual's sampling probability from the sampling strata. Analyses that are descriptive of the sample attributes are not weighted. Analyses that are descriptive of the population are weighted and are labeled as such. All logistic regression models used weights to account for the probability of selection by age and sex strata. Separate analyses were conducted for females and males. Due to gender differences in age of life events (e.g. marriage), females were age-group adjusted by 13-19 and 20-34 years and males by 13-24 and 25-34 years. Because of the strong association between HIV infection and marriage, bivariate and multivariate analyses were conducted separately for sexually active participants and those who had ever been married. Mutlivariate analyses were conducted with SAS's Proc SurveyLogistic.

Marriage was defined as a legal, religious, or customary agreement or a man and woman living together as married. Having ever been married included those who were currently married, separated, divorced, or a widow/widower. Anyone who had ever had sexual intercourse was considered sexually active. Polygamy, widow inheritance, and cultural rituals involving sex are part of Luo culture. [11]-[14] Ritual sex was defined as engaging in sexual intercourse to abide by cultural practices and beliefs.

We evaluated factors associated with HIV infection through logistic regression modeling using software that incorporated the sampling weights. Using the forward stepwise selection method, four hierarchical models were constructed: ever sexually active females and males and ever-married females and males. Each model included all covariates with a *p-value* ≤0.2 on bivariate analysis. First, sociodemographic variables were entered into the model beginning with the most significant covariate on bivariate analysis. Upon finalization of the sociodemographic tier, the same approach was used to add sexual behavior, other HIV risk factors, and finally STI-related covariates. Once a covariate was significant (*p-value* ≤0.05) within its tier, it was retained in the final model. Because STI-related covariates affected most models, we present the four models with and without STI covariates. The likelihood ratio test was used to compare statistical models. Although not significant on bivariate analyses, male circumcision was included in all male models because of the protection conferred against HIV. [15]-[17] Because of co-linearity between marriage and age group, “having ever been married” was not a covariate in the “ever sexually active” models for females and males.

## Results

Of 3960 randomly selected individuals, 2606 (66%) were eligible for enrollment; almost all others, 1261/1354 (93%), were ineligible because they had left Asembo. Of the eligible 2606, 1822 (70%) enrolled, 447 (17%) refused, and 337 (13%) consented at home but did not come to the study site. Enrollment rates were similar for males and females (70%) but higher among those aged <20 years (75%) than those aged ≥20 years (63%, *p*<0.0001). Primary reasons for refusal included not wanting blood taken (37%), partner/parental objection (19%), and not wanting to be tested for or learn one's HIV status (18%).

Among 13-19 year olds in the sample, 43.9% of females and 50.2% of males reported they had ever had sexual intercourse. The median age of sexual debut was 16.5 years for females and 15.5 years for males. The median age of first pregnancy was 18 years. The median age of first marriage was 18 years for females and 23 years for males.

Among currently married participants in the sample, 72/328 (22.0%) of females and 8/189 (4.2%) of males, were in polygamous marriages. Additionally, 63.9% (39/61) of widows had been inherited, and 2.8% (6/214) of ever-married men had inherited a widow. Of sexually active participants, 102/1202 (8.4%) had participated in ritual sex. Only 13.2% (77/583) of sexually active males were circumcised. There was high mobility with only 18% of female and 48% of male sexually active participants having lived their entire lives in Asembo.

The median number of lifetime sexual partners for sexually active participants in our sample was 3 for females and 4 for males. Over half of sexually active females, 327/619 (52.8%), had sex in exchange for gifts; 45/619 (7.3%) reported having ever been forced to have sex. Condom use during the last sexual intercourse was equally low between never-married males and females (25%). Among ever-married participants, condom use during the last sexual intercourse with spousal or non-spousal partners was rare in females (3.3%) and males (6.9%). Of currently married individuals, 3.7% (12/328) of females and 23.8% (45/189) of males reported having had sex with a non-spousal partner in the previous six months.

**Figure 1:**
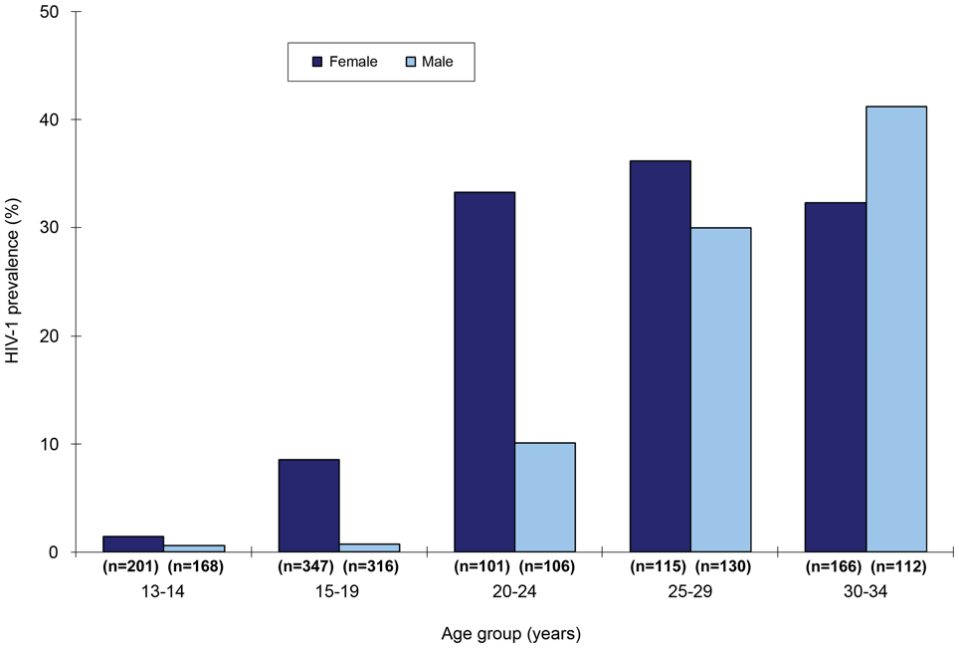
HIV prevalence by sex and age-group, N = 1762.

**Figure 2:**
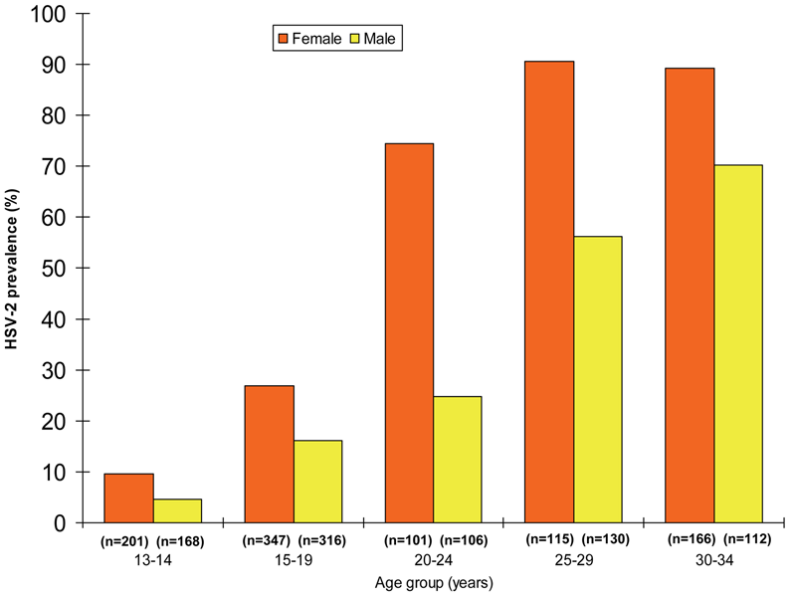
HSV-2 prevalence by sex and age group, N = 1762.

The population, HIV prevalence for the study area, based on 1762 participants who provided blood specimens and complete data, weighted by age group and sex was 15.4% overall, 20.5% among females, and 10.2% among males. Table 1 presents HIV prevalence of all study participants by demographic characteristics. Females became infected several years younger than males, and HIV prevalence was higher among females than males until the fourth decade. (Figure 1) Weighted HSV-2 prevalence was 40.0% overall, 53.0% among females, 25.8% among males, and remained higher in females for all age groups. (Figure 2) Although the study was not powered for single year of age-specific estimates, HIV prevalence in adolescent females increased from 3.1% [95% Confidence Intervals (CI), 0.0, 6.3] at 16 years to 12.8% (95% CI, 5.7, 19.9) at age 17.

**Table 1:** Demographic characteristics by weighted HIV prevalence and gender, N = 1762.

	Females				Males			
	N	HIV positive (n)	Weighted HIV prevalence (%)	95% CI^a^	N	HIV positive (n)	Weighted HIV prevalence (%)	95% CI
**Total**	930	162	20.5	[17.8, 23.2]	832	99	10.2	[8.5, 11.9]
**Age (years)** median = 19	median = 20				median = 18			
IQR^b^ = [15], [25]	IQR = [16], [26]				IQR = [15], [23]			
**Age group** **(years)**								
13-14	201	3	1.5	[0.0, 3.1]	168	1	0.6	[0.0, 1.7]
15-19	347	29	8.6	[5.7, 11.4]	316	2	0.8	[0.0, 1.8]
20-24	101	34	33.7	[24.7, 42.7]	106	11	10.4	[4.7, 16.1]
25-29	115	42	36.5	[27.9, 45.1]	130	39	30.0	[22.5, 37.5]
30-34	166	54	32.5	[25.8, 39.3]	112	46	41.1	[32.4, 49.7]
**Education**								
Some primary school	540	74	19.9	[16.9, 25.5]	409	26	5.9	[3.8, 8.1]
Completed primary school	239	46	22.7	[17.0, 28.5]	237	40	14.1	[10.1, 18.0]
Beyond primary school	151	28	18.9	[12.4, 25.4]	186	33	14.4	[9.9, 18.9]
**Occupation**								
Has cash income^c^	218	84	39.9	[33.2, 46.6]	219	55	21.9	[16.7, 27.0]
Employed	381	129	35.2	[30.2, 40.2]	354	90	21.8	[17.9, 25.7]
Unemployed	117	23	21.3	[13.5, 29.1]	51	4	7.5	[0.3, 14.8]
Student	428	9	2.1	[0.8, 3.5]	427	5	1.2	[0.2, 2.2]
**Religion**								
Muslim groups/Other	163	36	28.7	[21.2, 36.2]	154	16	8.0	[4.5, 11.5]
Protestant groups	539	95	19.8	[16.2, 23.3]	457	60	11.7	[9.0, 14.3]
Catholic groups	226	30	15.8	[10.6, 21.0]	221	23	8.6	[5.3, 11.8]
**Marital status**								
Never married	533	22	4.6	[2.8, 6.5]	618	24	3.8	[2.3, 5.3]
Currently married	328	92	29.4	[24.3, 34.6]	189	65	32.4	[26.0, 38.9]
Polygamous marriage	72	24	35.6	[23.9, 47.3]	8	2	24.6	[0.0, 53.0]
Divorced/Separated	6	2	36.2	[0.0, 76.6]	16	5	31.3	[9.7, 53.0]
Widow/Widower	61	45	77.8	[67.7, 88.0]	9	5	49.7	[17.1, 82.2]
**Pregnancy**								
Has ever been pregnant	433	138	33.2	[28.5, 37.8]	---	---	---	---
Currently pregnant	124	30	26.8	[18.6, 35.0]	---	---	---	---
**Sexual activity**								
Has had sexual intercourse	619	157	27.7	[24.1, 31.4]	583	97	14.1	[11.6, 16.5]
Has never had sexual intercourse	309	4	1.4	[0.1, 2.7]	249	2	0.8	[0.0, 1.8]

a 95% CI, 95% confidence intervals.

b IQR, interquartile range.

c Regular or intermittent employment that provided cash income such as salaried workers, small business owners, casual laborers, etc.

Of 309 females who denied ever having had sexual intercourse in our sample, 4 (1.3%) were HIV-positive and 5 (1.6%) were pregnant; 2/249 (0.8%) males who denied having had sexual intercourse were HIV-positive.

After adjustment for age group and stratification by sex, several demographic and risk variables were associated with HIV prevalence among sexually active participants (Table 2). Among sexually active females, HIV infection was significantly associated with the following factors: cash income through regular/intermittent employment, being married or widowed, higher number of lifetime sexual partners, ritual sex, having received an injection in the previous six months, scarification, HSV-2 infection, and past treatment for an STI. In multivariate analysis, HIV infection remained significantly associated with the following factors: older age, cash income, higher number of lifetime sexual partners, having received an injection in the prior six months, and scarification. Adding STI-related covariates to the model attenuated the positive association of HIV infection with age and lifetime number of sexual partners and rendered non-significant the positive association between HIV infection and scarification or prior STI treatment. HSV-2 infection remained strongly associated with HIV infection.

**Table 2:** Age-group adjusted risk factors associated with HIV infection by gender among sexually active participants, N = 1202.

		Females					Males				
		N	Weighted HIV prevalence (%)	Age group aOR^a^	95% CI^b^	*P-value*	N	Weighted HIV prevalence (%)	Age group aOR	95% CI	*P-value*
**Total**		619	27.7	4.2	[2.7, 6.6]	<0.001	583	14.1	11.8	[6.4, 21.6]	<0.001
**Age (years)**, overall median = 21		median = 22					median = 20				
IQR^c^ = [18], [28]		IQR = [18], [28]					IQR = [17], [26]				
**Demographic characteristics**											
*Education*											
Some primary school		316	30.1	*ref* d	–	NS^e^	230	9.5	*ref*	–	NS
Completed primary school		186	27.6	0.8	[0.5, 1.0]		190	17.6	1.1	[0.6, 2.0]	
Beyond primary school		117	22.2	0.6	[0.5, 1.2]		163	16.4	1.1	[0.7, 2.4]	
*Occupation*											
Has cash income^f^		213	40.7	2.1	[1.4, 3.1]	<0.001	215	22.3	1.2	[0.7, 2.0]	NS
*Religion*											
Muslim groups/other		117	36.2	*ref*	–	NS	109	11.2	*ref*	–	NS
Protestant groups		374	25.9	0.8	[0.4, 1.4]		321	16.4	1.6	[0.9, 3.0]	
Catholic groups		128	25.2	0.8	[0.5, 1.2]		153	11.1	1.0	[0.5, 2.1]	
*Marital status*											
Never married		224	8.6	*ref*		<0.001	369	5.6	*ref*	–	<0.05
Currently married		328	29.4	3.4	[1.5, 7.7]		189	32.4	2.4	[1.1,5.3]	
Divorced/Separated		6	36.2	5.0	[0.8, 21.0]		16	31.3	1.7	[0.5, 5.9]	
Widow/Widower		61	77.8	28.5	[10.6, 76.5]		9	49.7	5.2	[1.4, 19.7]	
*Mobility*											
Never lived outside Asembo		111	11.4	0.6	[0.3, 1.2]	NS	278	12.8	1.0	[0.6, 1.6]	NS
Spent >1 night away from home in previous 6 months		197	34.0	1.4	[1.0, 2.1]	NS	262	17.0	1.3	[0.7, 2.1]	NS
**Sexual behavior characteristics**											
Number of lifetime sexual partners for:											
Females: 1	Males: 1-3	144	9.2	ref		<0.001	238	3.1	ref		<0.001
2-4	4-9	394	28.0	2.3	[1.2, 4.8]		210	15.5	2.6	[1.2, 5.7]	
≥5	≥10	80	52.0	6.2	[2.7, 13.9]		129	33.4	5.4	[2.4, 12.1]	
Used a condom at last sexual intercourse		68	11.6	0.5	[0.2, 1.1]	NS	111	11.6	1.3	[0.7, 2.6]	NS
First partner >5 years older		152	32.0	1.1	[0.7, 1.7]	NS	---	---	---	---	
Ever had sex in exchange for gifts		327	26.6	0.9	[0.6, 1.3]	NS	---	---	---	---	
Sexual debut outside Asembo		57	25.6	0.8	[0.4, 1.6]	NS	78	23.3	1.7	[0.9, 3.2]	NS
Ritual sex^g^		24	49.6	3.8	[2.0, 7.5]	<0.01	78	20.2	1.9	[0.9, 4.0]	NS
Has ever been pregnant		433	33.2	1.8	[0.9, 3.6]	NS	---	---	---	---	
Currently pregnant		120	27.5	0.9	[0.5, 1.5]	NS	---	---	---	---	
**Other risk characteristics**											
Received injection in previous 6 months		249	35.7	1.8	[1.2, 2.6]	<0.01	209	16.9	1.8	[1.1, 2.9]	<0.05
Ever had scarification		220	34.8	1.7	[1.1, 2.5]	0.01	213	17.9	1.3	[0.8, 2.2]	NS
Alcohol intake during previous month		38	34.3	1.4	[0.6, 3.0]	NS	170	24.0	1.6	[0.9, 2.6]	NS
Circumcised		---	---	---	---		77	14.4	0.8	[0.4, 1.6]	NS
**Sexually transmitted infections (STI)**											
Herpes Simplex Virus-2 (HSV-2) infection		405	36.9	5.3	[2.7, 10.7]	<0.001	210	34.6	6.4	[3.2, 11.6]	<0.001
Previous treatment for an STI		41	46.2	2.1	[1.0, 4.2]	<0.05	130	43.8	5.4	[3.0, 9.9]	<0.001

a aOR, age-group adjusted odds ratio. Females by 13-19 and 20-34 years; males by 13-24 and 25-34 years.

b 95% CI, 95% confidence intervals.

c IQR, interquartile range.

d *ref*, referrent group.

e NS, not significant.

f Regular or intermittent employment that provided cash income such as salaried workers, small business owners, casual laborers, etc.

g For never married, this includes ritual sex with anyone; for ever married, this includes ritual sex with non-spousal partner only.

Among sexually active males (Table 2), factors significantly associated with HIV infection included the following: being married or a widower, higher number of lifetime sexual partners, having received an injection in the previous six months, HSV-2 infection, and previous STI treatment. In multivariate analysis, older age and higher number of lifetime sexual partners remained significantly associated with HIV infection. STI-related covariates in the model attenuated the association of both age and number of sexual partners with HIV infection, but HSV-2 infection and prior STI treatment remained associated with HIV infection.

Marital status was an effect modifier necessitating separate analyses by gender and marital history. Because few never-married individuals were HIV-infected, we were unable to conduct adequate multivariate analyses in this group. For ever-married participants, univariate analyses of factors associated with HIV infection are presented in Table 3 and multivariate analyses in Table 4. In multivariate analyses of ever-married females, widowhood, cash income, having received an injection in the prior six months, and HSV-2 infection were significantly associated with HIV infection. For ever-married males, a higher number of lifetime sexual partners and HSV-2 infection was significantly associated with HIV infection.

**Table 3:** Age-group adjusted risk factors associated with HIV infection by gender among participants who have ever been married^a^, N = 609.

		Females					Males				
		N	Weighted HIV prevalence (%)	Age group aOR^b^	95% CI^c^	*P-value*	N	Weighted HIV prevalence (%)	Age group aOR	95% CI	*P-value*
**Total**		395	36.4	1.3	[0.7, 2.7]	NS^d^	214	33.1	3.2	[1.0, 9.7]	<0.05
**Age (years)**, overall median = 27		median = 26					median = 28				
IQR^e^ = [23], [30]		IQR = [22], [30]					IQR = [25], [31]				
**Demographic characteristics**											
*Education*											
Some primary school		196	40.3	*ref* f	–	NS	60	34.6	*ref*	–	NS
Completed primary school		127	33.4	0.7	[0.5, 1.1]		95	29.2	0.7	[0.4, 1.4]	
Beyond primary school		72	31.6	0.8	[0.5, 1.4]		59	37.6	1.0	[0.5, 2.0]	
*Occupation*											
Has cash income^g^		189	41.8	1.6	[1.0, 2.5]	<0.05	135	36.9	0.8	[0.4, 1.4]	NS
*Religion*											
Muslim groups/Other		87	43.4	*ref*	–	NS	51	20.7	*ref*	–	NS
Protestant groups		236	34.4	0.8	[0.5, 1.4]		114	39.1	2.3	[1.1, 4.6]	
Catholic groups		72	34.1	0.8	[0.4, 1.5]		49	31.6	1.7	[0.7, 4.0]	
*Marital status*											
Currently married		328	29.4	*ref*	–	p<0.001	189	32.4	*ref*		NS
Polygamous marriage		72	35.6	1.5	[0.8, 2.7]	–	8	24.6	0.5	[0.1, 2.6]	
Divorced/Separated		6	36.2	1.4	[0.2, 8.4]		16	31.3	0.8	[0.3, 2.2]	
Widow/Widower		61	77.8	8.4	[4.4, 16.0]		9	49.7	2.1	[0.6, 7.1]	
*Mobility*											
Never lived outside Asembo		10	33.3	1.0	[0.2, 4.1]	NS	109	30.0	0.8	[0.5, 1.5]	NS
Spent >1 night away from home in previous 6 months		146	41.3	1.4	[0.9, 2.2]	NS	121	33.9	1.1	[0.6, 1.9]	NS
**Sexual behavior characteristics**											
Number of lifetime sexual partners for:											
Females: 1	Males: 1-3	26	21.8	*ref*		0.001	30	21.4	*ref*		<0.01
2-4	4-9	299	32.9	1.8	[0.7, 4.7]		96	24.5	1.0	[0.4, 2.4]	
>5	>10	69	55.2	4.4	[1.5, 12.9]		85	46.3	2.6	[1.1, 6.0]	
Age difference with first partner >5 years		131	34.8	0.9	[0.6, 1.4]	NS	18	16.1	0.4	[0.1, 1.2]	NS
If currently married, had extramarital sex in previous 6 months		12	32.9	1.2	[0.3, 4.3]	NS	44	33.3	1.1	[0.5, 2.4]	NS
Had ritual sex with non-spousal partner		11	94.2	29.6	[4.1, 213.6]	<0.05	24	38.2	1.2	[0.5, 3.2]	NS
Has ever been pregnant		386	36.0	0.7	[0.1, 3.2]	NS	---	---	---	---	
Currently pregnant		91	30.9	0.7	[0.4, 1.3]	NS	---	---	---	---	
**Other risk characteristics**											
Received injection in previous 6 months		180	44.4	1.9	[1.2, 2.9]	<0.01	76	43.6	2.2	[1.2, 3.9]	<0.01
Ever had scarification		157	42.6	1.5	[1.0, 2.4]	NS	93	32.8	1.1	[0.6, 1.9]	NS
Alcohol intake during previous month		23	45.8	1.5	[0.6, 3.7]	NS	91	33.0	1.0	[0.5, 1.7]	NS
Circumcised		---	---	---	---	---	35	23.6	0.5	[0.2, 1.1]	NS
**Sexually transmitted infections (STI)**											
Herpes Simplex Virus-2 (HSV-2) infection		341	39.8	3.5	[1.6, 7.7]	0.001	139	44.0	3.8	[1.9, 7.7]	<0.001
Previous treatment for an STI		31	48.3	1.7	[0.8, 3.7]	NS	96	45.5	2.5	[1.4, 4.4]	<0.01

a Includes currently married, separated, divorced, or widowed.

b aOR, age-group adjusted odds ratio. Females by 13-19 and 20-34 years; Males by 13-24 and 25-34 years.

c 95% CI, 95% confidence intervals.

d NS, not significant.

e IQR, interquartile range.

f *ref*, referrent group.

g Regular or intermittent employment that provided cash income such as salaried workers, small business owners, casual laborers, etc.

**Table 4:** Logistic regression risk factor analysis models for HIV infection among self-reported sexually active participants.

		All sexually active								Ever married^a^							
		Females (n = 593)				Males (n = 534)				Females (n = 383)				Males (n = 216)			
		Model I		Model I + STI^b^		Model II		Model II + STI		Model III		Model III + STI		Model IV		Model IV + STI	
		aOR^c^	95% CI^d^	aOR	95% CI	aOR	95% CI	aOR	95% CI	aOR	95% CI	aOR	95% CI	aOR	95% CI	aOR	95% CI
**Demographic co-variates**																	
Older age group^e^		2.1	[1.2, 3.6]	1.4	[0.8, 2.6]	7.5	[3.6, 15.9]	3.7	[1.6, 8.5]	1.0	[0.5, 2.2]	0.8	[0.4, 1.7]	3.1	[1.0, 9.1]	2.0	[0.6, 7.1]
Widow/widower		–f	–	–	–	–	–	–	–	7.6	[3.8, 15.4]	7.5	[3.8, 15.0]	–	–	–	–
Has cash income^g^		2.0	[1.3, 3.1]	1.9	[1.2, 3.0]	–	–	–	–	1.6	[1.0, 2.7]	1.6	[1.0, 2.7]	–	–	–	–
Muslim-based religious group member		–	–	–	–	–	–	–	–	–	–	–	–	0.5	[0.2, 1.2]	0.5	[0.2, 1.3]
**Sexual risk behavior co-variates**																	
Number of lifetime sexual partners for:																	
Females	Males																
1	1-3	*ref* h		*ref*		*ref*		*ref*		*ref*		*ref*		*ref*		*ref*	
2-4	4-9	2.1	[1.0, 4.5]	1.4	[0.7, 3.1]	2.8	[1.2, 6.8]	1.8	[0.8, 4.4]	1.3	[0.5, 3.6]	1.1	[0.4, 3.2]	1.4	[0.5, 3.5]	1.0	[0.4, 2.3]
≥5	≥10	5.6	[2.4, 2.8]	3.4	[1.4, 8.5]	6.6	[2.6, 16.9]	3.6	[1.3, 9.9]	2.7	[0.9, 8.5]	2.1	[0.7, 6.8]	3.2	[1.3, 8.1]	2.3	[1.0, 5.9]
**Other HIV risk co-variates**																	
Received injection in previous 6 months		1.8	[1.2, 2.8]	1.9	[1.2, 2.9]	–	–	–	–	2.1	[1.3, 3.4]	2.1	[1.3, 3.4]	–	–	–	–
Ever had scarification		1.6	[1.1, 2.5]	1.6	[1.0, 2.4]	–	–	–	–	–	–	–	–	–	–	–	–
Circumcised		N/A^i^	N/A	N/A	N/A	0.7	[0.3, 1.4]	0.6	[0.3, 1.3]	N/A	N/A	N/A	N/A	0.7	[0.3, 1.8]	0.5	[0.2, 1.5]
**STI co-variates**																	
Herpes Simplex Virus-2 (HSV-2) infection		N/A	N/A	4.1	[1.9, 8.7]	N/A	N/A	5.1	[2.5, 10.4]	N/A	N/A	3.4	[1.3, 8.8]	N/A	N/A	4.3	[2.0, 9.2]
Previous treatment for an STI		N/A	N/A	–	–	N/A	N/A	2.3	[1.1, 4.7]	N/A	N/A	–	–	N/A	N/A	–	–

a Includes currently married, separated, divorced, or widowed.

b STI, sexually transmitted infections.

c aOR, adjusted odds ratio.

d 95% CI, 95% confidence intervals.

e Age groups: Females by 13-19 and 20-34 years; Males by 13-24 and 25-34 years.

f A co-variate that was not included in the final model.

g Regular or intermittent employment that provided cash income such as salaried workers, small business owners, casual laborers, etc.

h *ref*, referrent group.

i N/A, not applicable

Limited data were available on circumstances around injections. Of 458 sexually active individuals who reported receiving an injection in the previous six months in our sample, 87% received it from clinicians, 9% from community health workers, and 4% from traditional healers/herbalists. Those aged 30-34 years comprised the highest proportion receiving an injection (42.1%); 15-19 year olds were the lowest (35.2%).

## Discussion

This population-based study in rural Nyanza Province, Kenya, found a high prevalence of HIV infection among those aged 13-34 years. Despite a decreasing trend in Kenya's national HIV prevalence, [18] similarly high rates were reported in the 2003 KDHS for Nyanza Province [3] and the Kenya AIDS Indicator Survey 2007. [2] In our study, HIV prevalence among females aged 15-19 years was 12 times higher than their male counterparts. This substantial gender disparity is found in Kenya nationwide [3], [19] and other African countries. [6], [18], [20] Social [21]-[24] and biological factors may explain this difference. [25] Young women may have sex with older, more sexually experienced men, who are more likely to be HIV-infected. [26] Young women in our study, however, described most of their sexual experiences to be with peers. Having a first sexual partner much older in age was not significantly associated with HIV infection. [27] Biological factors including immature genital tracts, cervical ectopy, [28] and more efficient male-to-female HIV transmission [29] likely contribute to the vulnerability of young women to HIV infection.

HSV-2 may also play a role. The strong association we found between HIV and HSV-2 infection confirms findings from other studies. [7], [30]-[37] A meta-analysis of longitudinal studies with established temporal relationships between HIV and HSV-2 acquisition found that HSV-2 infection increases the risk for HIV acquisition three-fold. [31] HSV-2 infection could be a driving force for HIV transmission in our study population.

Including HSV-2 infection in the multivariate model attenuated the positive association between HIV and a higher lifetime number of sexual partners. Although under-reporting of lifetime number of sexual partners is a possible explanation, HSV-2 infection, particularly in young people, may be a more accurate marker of sexual activity than reported sexual behavior. [7] Because HSV-2 infection is such a strong co-factor in sexual transmission of HIV, differences in lifetime number of sexual partners, depending on HIV prevalence among such partners, may translate into relatively small differences in risk for HIV infection in the presence of HSV-2 infection.

Marriage has been associated with risk for HIV infection in sub-Saharan Africa. [3], [7] In our study, widowhood was strongly associated with HIV infection, presumably because many widows lost their husbands to AIDS. Being currently married was significantly associated with HIV infection. HIV prevention efforts must target married couples. The 2003 KDHS found 17.1% of couples in Nyanza Province to be HIV discordant and 9.8% concordantly HIV-infected. [3] The low frequency of condom use within marriage and the high frequency of extramarital sex among men in our study suggest that a substantial proportion of married people in this community will be exposed to HIV infection through unprotected sexual intercourse with their HIV-infected spouse.

The potential contribution of unsafe injections to HIV transmission has been raised [38]-[42], disputed [43]-[45], and continues to be examined. [46] In our study, having received an injection in the prior six months was associated with HIV infection for sexually active or ever-married females but not for males or never-married participants. Additionally, for all participants there was no association between having received an injection in the prior six months and having ever had STI treatment. Given this study's cross-sectional design, this finding may represent reverse causality or confounding for which we could not control. Further investigation through a prospective incidence cohort is warranted.

HIV infection in Africa has been associated with urban-to-rural and intra-rural human mobility. [47]-[49] Females in our study reported more mobility than males; HIV prevalence was lower among females and males who had lived their entire lives in Asembo than those who had spent more than one night away from home in the previous six months. Mobility may be a risk factor for HIV infection. The 2002 DSS data for Asembo showed substantial migration. In-migration exceeded out-migration, adults younger than 30 years were the most migratory, and women migrate more than men. [5] Although primary reasons for migration included marriage and seeking employment, health-related reasons cannot be ruled out. [5] Further investigation is warranted to better elucidate the roles of mobility and migration in Asembo's HIV epidemic.

Recent randomized clinical trials of male circumcision resulted in an approximate reduction of HIV acquisition by half. [15]-[17] Luo males are not routinely circumcised, [3], [50] which could explain the higher HIV prevalence in Asembo compared to non-Luo areas of Nyanza Province and Kenya. [3], [51]

Ritual sex around funerals, polygamy, and widow inheritance are traditional Luo practices that can facilitate HIV transmission. [11]-[14] The low rates of reported ritual sex participation in this study may reflect a decreasing prevalence of such practices or underestimate their relevance, due to the young study population. Traditionally, widow inheritance requires a widow to become the wife of her late husband's brother, assuring her and her children economic and social stability. [11]-[14] Although we lacked sufficient power to detect associations between HIV infection and polygamy, widow inheritance, or ritual sex, the strong association between HIV infection and widowhood and the high prevalence of inheritance among widows in our study suggest that widow inheritance could play a role in this epidemic. [11]-[14]

Other data regarding HIV prevalence in Nyanza Province include a second cross-sectional study with different sampling methodology and age distribution was conducted from 2004-2005 in the neighboring community of Gem. This study of volunteers aged 15-34 years showed a weighted HIV prevalence of 28.2% among females and 14.8% among males. [52] Cohen *et al*. present HIV prevalence data from a 2006 general population survey in Kisumu city, the capital of Nyanza Province, conducted to evaluate antiretroviral therapy-related attitudes and beliefs. Although an urban setting, they found a similar over HIV prevalence of 25% in women and 16% in men aged 15-49 years. [53]

Study limitations exist. Risk factor data in cross-sectional studies are associations and cannot identify causality. There is potential for selection bias from migration, because 30% of selected individuals had moved out of Asembo between the DSS survey round and study enrollment (<8 months). Ill individuals may have left the area seeking health care or returned home to die. Healthy individuals may have left seeking employment or returned to care for ill relatives. By requiring participants to visit a nearby site, we may have selected for healthier individuals. Since study participation took up to four hours, we may have selected for individuals who were wealthier, unemployed, or had time. As the first HIV-specific research conducted in Asembo, individuals with self-perceived low HIV risk may have self-selected to participate. Most of these potential biases would have resulted in lower HIV prevalence estimates. However, if the individuals aged ≥20 years who refused study participation did so because they engaged in risky sexual behavior and did not want to know their HIV status, their non-participation might have resulted in an underestimation of HIV prevalence.

Recall bias due to illness, gender, or age can occur when describing sexual histories or risk behaviors. Sexual behavior interviews are limited by the accuracy of self-reporting, and women, particularly adolescent females, frequently underreport sexual experiences. [25]

### Conclusion

Our study shows a large HIV-burden in this area of rural Nyanza. High HIV transmission rates are probable given the rapid increase in age-specific HIV prevalence among adolescent females. Without massive and drastic prevention and treatment efforts, AIDS will continue to devastate Asembo. Community-wide delivery of HIV counseling and testing with HIV/AIDS treatment provision merits exploration. Alterations in cultural perceptions and practices are needed to delay sexual debut, reduce the number of sexual partners, promote mutual faithfulness to known seronegative partners, implement 100% condom use with casual partners, and discourage inter-generational and transactional sex. Community elders and opinion leaders are urged to encourage non-sexual substitutions for ritual sex and empower widows refusing to be inherited. In an area with such high HIV prevalence, key life decisions should be made based on known HIV serostatus.

Qualitative data are required to better elucidate circumstances around individual HIV transmission/acquisition events and identify behaviors driving the epidemic in this area. Interventions to achieve high male circumcision rates are needed along with continued research on additional HIV prevention strategies including HIV vaccines, microbicides, pre-exposure chemoprophylaxis, and vaccine and chemotherapeutic agents against HSV-2.
